# CDK4/6 inhibitors for hormone receptor-positive/human epidermal growth factor receptor 2 negative advanced breast cancer: A rapid health technology assessment

**DOI:** 10.1097/MD.0000000000035487

**Published:** 2023-10-06

**Authors:** Hangye Gu, Yaqing Chen, Zeyu Xie, Yong Chen

**Affiliations:** a Key Specialty of Clinical Pharmacy, the First Affiliated Hospital of Guangdong Pharmaceutical University, Guangzhou, China.

**Keywords:** advanced breast cancer, CDK4/6 inhibitors, drug selection, quantitative scoring, rapid health technology assessment

## Abstract

**Object:**

Based on the best available evidence, rapid health technology was used to assess 4 CDK4/6 inhibitors approved for marketing in China. This assessment aims to provide a reference basis for the selection of drugs by medical institutions in China and to promote the rational use of drugs in the clinic.

**Methods:**

Depending on the *Rapid Guidelines for Drug Evaluation and Selection in Chinese Medical Institutions (the Second Edition*), a percentage quantitative scoring approach was used to objectively score the pharmacological properties, efficacy, safety, economy, and other attributes of CDK4/6 inhibitors.

**Results:**

The composite score rankings were, in descending order, 78.09 points for abemaciclib, 78.04 points for palbociclib, 72.15 points for dalpiciclib, and 69.24 points for ribociclib by integrating the result of the 5 dimensions.

**Conclusion:**

Until the clinical studies, guideline recommendations, prices, and many other aspects of this assessment are updated, abemaciclib and palbociclib, which have the top 2 scores, can be used as a priority recommendation for Chinese medical institutions to select CDK4/6 inhibitors and optimize the use of the drug catalog based on the scoring results of this assessment.

## 1. Introduction

Breast cancer is the malignant tumor with the highest incidence rate and the highest number of deaths among women worldwide, seriously threatening women’s physical and mental health.^[1]^ Hormone receptor-positive/human epidermal growth factor receptor 2-negative (HR+/HER2−) breast cancers account for up to 70% of all breast cancers.^[2]^ The main first-line therapeutic strategies for patients with HR+/HER2− breast cancers are chemotherapy and endocrine therapy (ET). With a long median remission period, low toxicity, and fewer adverse effects than chemotherapy, ET has become the first-line standard of care for patients with HR+/HER2− breast cancer. However, with the widespread use of endocrine drugs, the problem of drug resistance is gradually emerging. About one-quarter of patients with HR+/HER2− breast cancer that develops resistance will experience recurrence or metastasis, leading to the stage of incurable advanced or metastatic breast cancer.^[3]^ In recent years, cell cyclin-dependent kinase 4/6 (CDK4/6) inhibitors have been reported to be an effective treatment for ET-resistant HR+/HER2− breast cancer, whose introduction has changed the treatment modality for HR+/HER2− breast cancer patients.^[4]^ Currently, CDK4/6 inhibitors combined with ET have been used as the preferred treatment strategy for HR+/HER2− advanced breast cancer patients in guidelines such as *NCCN Guidelines for Breast Cancer, Version 4.2023*,^[5]^ and *CSCO Guidelines for the Treatment of Breast Cancer 2023*.^[6]^

CDK4/6 inhibitors are critical kinases involved in cell cycle regulation. They can effectively inhibit tumor cell proliferation, overcome or delay the emergence of endocrine resistance, and strive for longer patient survival time.^[7,8]^ To date, a total of 4 CDK4/6 inhibitors have been approved for marketing in China for the treatment of HR+/HER2− locally advanced or metastatic breast cancer, namely palbociclib, ribociclib, abemaciclib, and dalpiciclib. Although these 4 CDK4/6 inhibitors have similar mechanisms of action, they still differ in efficacy, safety, price, and accessibility.

Rapid Health Technology Assessment is an emerging methodology that allows for the rapid integration of existing evidence, and is characterized by its rapidity, comprehensiveness, science, objectivity and impartiality. Decision makers can use rapid health technology assessment to objectively select and evaluate medicines entering and leaving healthcare facilities according to the actual needs of healthcare facilities.

This assessment is based on the *Rapid Guidelines for Drug Evaluation and Selection in Chinese Medical Institutions(the Second Edition*) (referred to as the “*Rapid Guidelines*”).^[9]^ It provides an objective quantitative scoring of the pharmacological properties, efficacy, safety, economy, and other attributes of CDK4/6 inhibitors, intending to provide a reference basis for China’s medical institutions in the selection of drugs and optimization of the drug catalog and promoting the rational use of drugs in the clinic.

## 2. Methods

### 2.1. Drugs

Drugs to be assessed are 4 original CDK4/6 inhibitors approved for marketing in China, including palbociclib (125 mg, Pfizer Manufacturing Deutschland GmbH, Betriebsstatte Freiburg, Germany), ribociclib (200 mg, Novartis Singapore Pharmacological Manufacturing Pte. Ltd., Singapore), abemaciclib (150 mg, Lilly del Caribe Inc., carolina, puerto rico), dalpiciclib (150 mg, Jiangsu Hengrui Pharmaceuticals CO., LTD, Jiangsu, China). Specification selection is based on commonly used specifications in clinical practice.

### 2.2. Materials and methods

We searched Chinese and English databases such as CNKI, Wanfang, VIP, SinoMed, PubMed, The Cochrane Library, Embase, and other official websites such as NMPA, WHO, FDA, National Medical Security Administration, and corporate websites to review relevant literature, drug instructions, and drug registration materials. To collect data on the pharmacologic effects, in vivo processes, indications, guideline recommendations, clinical efficacy, adverse effects, and price of 4 CDK4/6 inhibitors. Then based on the bylaw scoring criteria of the Rapid Guidelines, a percentage quantitative scoring method was used to provide objective quantitative scores for the 5 dimensions of CDK4/6 inhibitor pharmacological properties, efficacy, safety, economy, and other attributes.

## 3. Results

### 3.1. Pharmacological properties score

#### 3.1.1. Pharmacological effects and in vivo processes.

The 4 CDK4/6 inhibitors are all critical kinases involved in cell cycle regulation, which can reduce the phosphorylation level of retinoblastoma protein (Rb) downstream of the CDK4 and 6 signaling pathway, prevent the progression of the cell cycle from the G1 phase to S phase, and regulate the cell cycle from the source position to achieve the purpose of inhibiting the proliferation of tumors.^[10–12]^ At the same time, CDK4/6 inhibitors can inhibit the expression of the upstream estrogen receptor signaling pathway, and there is a synergistic effect with endocrine therapy to achieve the delay and reversal of endocrine drug resistance.^[13,14]^ Based on these excellent pharmacological effects, authoritative guidelines in China and abroad have identified CDK4/6 inhibitors in combination with ET as the preferred treatment strategy for patients with HR+/HER2− advanced breast cancer. To summarize, 4 CDK4/6 inhibitors have a definite clinical curative effect, precise and innovative mechanism of action, and all scored 5 points. Furthermore, all 4 CDK4/6 inhibitors scored 5 points for clear in vivo processes and completed pharmacokinetic parameters by reviewing their respective instructions.

#### 3.1.2. Pharmacy and usage.

In the instruction manual, the main ingredients and excipients of palbociclib, ribociclib, and abemaciclib are straightforward, gettting 2 points. The main ingredients of dalpiciclib are straightforward, but the excipients are unclear, so get 1 point. When used in combination with ET for HR+/HER2− breast cancer, the recommended doses per dose of palbociclib, ribociclib, abemaciclib, and dalpiciclib are 125 mg, 600 mg, 150 mg, and 150 mg, respectively. Among them, palbociclib has 3 specifications of 75 mg, 100 mg, and 125 mg. Ribociclib has only one specification of 200mg. Abemaciclib has 3 specifications of 75 mg, 100 mg, and 150 mg. Furthermore, dalpiciclib has 3 specifications of 50 mg, 125 mg, and 150 mg. The specifications of the 4 CDK4/6 inhibitors can all meet the clinical application and dose adjustment, scoring 2 points.

All 4 CDK4/6 inhibitors are oral formulations and can be self-administered without assistance. Moreover, the recommended doses of the 4 CDK4/6 inhibitors are fixed, so there is no need to adjust the dose during use except for individualized factors such as severe or intolerable adverse reactions. Therefore, all 4 CDK4/6 inhibitors scored 2 points in 3 aspects: appropriate dosage form, administration dose, and ease of use. The recommended dosing frequency of palbociclib, ribociclib, and dalpiciclib is once a day, with a score of 2. Furthermore, the recommended dosing frequency of abemaciclib is twice a day, with a score of 1.5.

#### 3.1.3. Storage conditions and expiration date.

An instruction review shows that all 4 CDK4/6 inhibitors require sealed storage at room temperature, scoring 3 points. In addition, none of them mentioned in the instruction that they needed to be shielded from light, so this item scored 1 point. All 4 CDK4/6 inhibitors scored 1.5 points for having an expiration date of 36 months.

In summary, the pharmacological properties scores of palbociclib, ribociclib, abemaciclib, and dalpiciclib were 27.5, 27.5, 27, and 26.5, respectively, as shown in Table 1.

**Table 1:** Pharmacological properties score results.

Index system and weight coefficient	Details (indicator information and data sources)	Palbociclib	Ribociclib	Abemaciclib	Dalpiciclib
Pharmacological effect (5)	5 The clinical curative effect is definite, the mechanism of action is clear, and the mechanism or target of action is innovative	5	5	5	5
	4 Definite clinical efficacy and clear mechanism of action				
	2 The clinical efficacy is acceptable, but the mechanism of action is not yet clear				
	1 The clinical effect is average, and the mechanism of action is not clear				
In vivo process (5)	5 The in vivo process is clear and the pharmacokinetic parameters are complete	5	5	5	5
	3 The in vivo process is basically clear, and the pharmacokinetic parameters are incomplete				
	1 The in vivo process is not clear, no relevant pharmacokinetic studies				
Pharmacy and method of use (multiple choices available) (12)	2 The main ingredients and excipients are clear (all clear 2, one item clear 1)	2	2	2	1
	2 Specifications and packaging (all applicable to clinical application/dose adjustment 2, one appropriate 1)	2	2	2	2
	2 Appropriate dosage form (oral/inhalation/topical preparation 2, subcutaneous/muscular injection 1.5, intravenous drip/intravenous injection 1)	2	2	2	2
	2 Appropriate dosage form (oral/inhalation/topical preparation 2, subcutaneous/muscular injection 1.5, intravenous drip/intravenous injection	2	2	2	2
	2 Dosing frequency (≤qd, 2; bid, 1.5; ≥tid 1)	2	2	1.5	2
	2 convenient to use (Self-administered without assistance 2, Self-administer without assistance, with the help of others or after training 1.5, Medical staff administer medication 1, Patients need to be administered with the help of medical staff or by medical staff, and the medication requires special consumables or pretreatment 0.5)	2	2	2	2
Storage conditions (multiple choice) (4)	3 Storage at room temperature	3	3	3	3
	2 Store in the shade				
	1 Refrigerated/Freezer Storage				
	1 Need to be shaded or protected from light	1	1	1	1
Drug expiration date (2)	2 ≥ 60 months				
	1.5 ≥ 36 months, <60 months	1.5	1.5	1.5	1.5
	1 ≥ 24 months,<months				
	0.5 ≥ 12 months, <24 months				
	0.5 <12 months				
Pharmaceutical properties score		27.5	27.5	27	26.5

### 3.2. Efficacy score

#### 3.2.1. Indications.

Based on rich evidence-based medical evidence, authoritative in *NCCN Guidelines for Breast Cancer, Version 4.2023* and *CSCO Guidelines for the Treatment of Breast Cancer 2023*, and other authoritative guidelines, CDK4/6 inhibitors combined with ET are recommended as the first choice for patients with HR+/HER2− advanced breast cancer, so the 4 CDK4/6 inhibitors are all the first choice drugs that are clinically necessary, scoring 5 points.

#### 3.2.2. Guide recommendation.

This assessment aims to provide a reference for Chinese medical institutions to select CDK4/6 inhibitors. Based on the approved indications, accessibility, and other reasons, this item mainly refers to the *CSCO Guidelines for the Treatment of Breast Cancer 2023* for objective scoring.

Palbociclib and ribociclib are grade I recommendations (1A) in all stratifications of HR+/HER2− advanced breast cancer, scoring 12 points. After ET and Tamoxifen (TAM) therapy failed, aromatase inhibitor (AI) + ribociclib is a grade II recommendation (1A). Moreover, after the failure of non-steroidal AI therapy and the failure of steroidal AI therapy, fulvestrant + ribociclib is a class II recommendation (1B). This assessment is based on the data of the highest level of evidence for ribociclib class II recommendation (1A), scoring 9 points. After TAM treatment failure, AI + dalpiciclib is a grade II recommendation (1B). Furthermore, after non-steroidal AI therapy failure and steroid AI therapy failure, fulvestrant + dalpiciclib is a grade I recommendation (1A). This assessment is based on the data of the highest level of evidence for dalpiciclib class I recommendation, scoring 12 points. Guideline recommendations are detailed in Table 2.

**Table 2:** Recommendations for HR+/HER2− advanced endocrine rescue therapy in *CSCO Guidelines for the Treatment of Breast Cancer 2023.*

Layered	Grade I recommendation	Grade II recommendation	Grade III recommendation
Without endocrine therapy	AI + CDK4/6i (palbociclib, abemaciclib) (1A)	1. AI + ribociclib (1A) 2. fulvestrant + CDK4/6i (2A) 3. AI (2A) 4. fulvestrant (2A)	TAM (2B)
TAM treatment failure	AI + CDK4/6i (palbociclib, abemaciclib) (1A)	1. AI + chidamide (1A) 2. AI + ribociclib (1A) 3. AI + dalpiciclib (1B) 4. AI + everolimus (2A) 5. fulvestrant + CDK4/6i (1B)	1. AI (2A) 2. fulvestrant (2A)
Non-steroidal AI treatment failure	fulvestrant + CDK4/6i (palbociclib, abemaciclib, dalpiciclib ) (1A)	1. steroids AI + chidamide (1A) 2. fulvestrant + ribociclib (1B) 3. Steroids AI + everolimus (1B)	1. fulvestrant (2A) 2. steroids AI (2A) 3.TAM or toremifene (2B) 4. progestin (2B)
Steroids AI treatment failure	fulvestrant + CDK4/6i (palbociclib, abemaciclib, dalpiciclib) (1A)	1. fulvestrant + ribociclib (1B) 2. fulvestrant + everolimus (2A) 3. non-steroidal AI + CDK4/6i (2A)	1. fulvestrant (2A) 2. non-steroidal AI (2B) 3.TAM or toremifene (2B) 4. progestin (2B)
Failure of CDK4/6 inhibitor therapy	/	1. another CDK4/6i + endocrine (2A) 2. Other targeted drugs (like everolimus chidamide, alpelisib) + endocrine (2A) 3. Participate in rigorously designed clinical studies	1. progestin (2B) 2. toremifene (2B)

AI = aromatase inhibitor, TAM = tamoxifen.

#### 3.2.3. Clinical efficacy.

This assessment takes the median progression-free survival (PFS) as the primary efficacy indicator and median overall survival (OS) as a secondary efficacy indicator. In the latest data released by the trials of PALOMA-3 (Palbociclib),^[15]^ MONALEESA-3 (ribociclib),^[16]^ MONARCH-2 (abemaciclib),^[17,18]^ and DAWNA-1 (dalpiciclib),^[19]^ compared with placebo + fulvestrant, the median PFS was prolonged by 6.6 months, 5.5 months, 7.6 months and 8.5 months for palbociclib, ribociclib, abemaciclib and dalpiciclib in combination with fulvestrant, so that the scores of the primary efficacy indicators were 4 points, 3 points, 5 points, and 6 points, respectively. The median OS of palbociclib, ribociclib, and abemaciclib were prolonged by 6.8 months, 12.2 months, and 9.4 months, respectively. Dalpiciclib’s OS data is immature. Therefore, the scores of the secondary efficacy indicators of palbociclib, ribociclib, abemaciclib, and dalpiciclib were 2 points, 4 points, 3 points, and 1 point, respectively.

In summary, the efficacy scores of palbociclib, ribociclib, abemaciclib, and dalpiciclib were 23 points, 21 points, 25 points, and 24 points, respectively, as shown in Table 3.

**Table 3:** Efficacy score results.

Index system and weight coefficient	Details (indicator information and data sources)	Palbociclib	Ribociclib	Abemaciclib	Dalpiciclib
Indications (5)	5 clinically necessary, preferred	5	5	5	5
	3 Clinical needs, second choice				
	1 More medicines to choose from				
Guide recommendation (12)	12 Diagnosis and treatment norms/clinical pathways, consensus issued by national health administrative agencies/management measures, etc., guideline level I recommendation (A-level evidence 12, B-level evidence 11, C-level evidence and others 10)	12		12	12
	9 Guidelines level II and below recommendations (A-level evidence 9, B-level evidence 8, C-level evidence and others 7)		9		
	6 Expert consensus recommendation (consensus 6 issued by the society based on systematic reviews, consensus 5 issued by the society, and others 4)				
	3 Systematic review/Meta-analysis (large sample, high-quality systematic review/Meta-analysis 3, small sample, low quality systematic review/meta-analysis 2, systematic review/meta-analysis of non-RCT study 1)				
Clinical efficacy (10)	6 Score by primary efficacy endpoint	4	3	5	6
	4 Score by secondary efficacy endpoint	2	4	3	1
Effectiveness score		23	21	25	24

### 3.3. Safety score

#### 3.3.1. Adverse effect.

The adverse effects of the 4 CDK4/6 inhibitors are roughly similar, and the common (occurrence frequency ≥ 10%) adverse effects include neutropenia, diarrhea, infection, leukopenia, fatigue, nausea, liver toxicity, and so on. The most common adverse reaction was neutropenia. The incidence of grade 3 neutropenia with palbociclib, ribociclib, abemaciclib, and dalpiciclib were 64.6% to 68.4%, 53.4% to 65.0%,18.6% to 28.2%, 84.2% to 85.8%, respectively, all ≥ 10%.^[20]^ Therefore, the moderate and severe adverse reactions of the 4 CDK4/6 inhibitors all scored 1 point.

#### 3.3.2. Special groups.

At present, there is no relevant data to determine the safety and efficacy of the 4 CDK4/6 inhibitors in children and adolescents under 18, and their use is not recommended, with a score of 0 for all. Palbociclib, ribociclib, and abemaciclib do not require dose adjustment for elderly patients aged 65 and over, scoring 1 point. Dalpiciclib currently has limited application data in elderly patients aged 65 and over, and it is recommended to use it under the guidance of a doctor, scoring 0.5 points. None of the 4 CDK4/6 inhibitors is recommended for pregnant and lactating women; both items score 0 points.

For patients with mild or moderate liver damage, palbociclib and abemaciclib do not require dosage adjustment. For patients with severe hepatic impairment, it is recommended to reduce the dose of palbociclib to 75 mg per dose and reduce the frequency of abemaciclib to once a day, scoring 3 points. There is no need to adjust the dose of ribociclib for patients with mild hepatic impairment, and it is recommended to reduce the dose to 400 mg per dose for patients with moderate and severe hepatic impairment, scoring 3 points. Moreover, there is no pharmacokinetic study of dalpiciclib in patients with hepatic impairment, and it is not recommended to use dalpiciclib in patients with moderate or severe hepatic impairment, so a score of 1 is awarded.

No dose adjustment is required for palbociclib in patients with mild, moderate, or severe renal impairment, scoring 3 points. Ribociclib and abemaciclib do not require dose adjustment in patients with mild or moderate renal impairment, and no studies of ribociclib or abemaciclib have been conducted in breast cancer patients with severe renal impairment, so all score 2 points. There are no studies of dalpiciclib in patients with renal impairment, and it is not recommended for patients with moderate or severe renal impairment, scoring 1 point.

#### 3.3.3. Drug interactions and other aspects.

The 4 CDK4/6 inhibitors are mainly metabolized by liver drug enzyme CYP3A4. CYP3A4 inhibitors such as clarithromycin, ketoconazole, and voriconazole can reduce the metabolism of CDK4/6 inhibitors, increase drug concentration, and increase the occurrence of adverse effects. CYP3A4 inducers such as phenytoin, carbamazepine, rifampin, and St. John’s wort can accelerate the metabolism of CDK4/6 inhibitors and affect the efficacy of CDK4/6 inhibitors. Therefore, all 4 CDK4/6 inhibitors should be avoided in combination with CYP3A4 inhibitors or CYP3A4 inducers, all scoring 1 point.

The common adverse effects of the 4 CDK4/6 inhibitors are reversible and can be recovered by reducing the dose or stopping the drug, scoring 1 point. None of the 4 CDK4/6 inhibitors have been studied for human carcinogenicity, and all scored 0 points. FDA, NMPA, and other government websites have not issued medication warnings for the 4 CDK4/6 inhibitors, all scoring 1 point.

In summary, the safety scores of palbociclib, ribociclib, abemaciclib, and dalpiciclib were 12 points, 11 points, 11 points, and 7.5 points, respectively, as shown in Table 4.

**Table 4:** Safety score results.

Safety (25)			Palbociclib	Ribociclib	Abemaciclib	Dalpiciclib
Adverse reactions (multiple choices) (8)	Moderate adverse reactions (3)	3 incidence rate < 1%	1	1	1	1
		2 incidence rate 1% to <10%				
		1 incidence rate > 10%				
		0 ADR occurrence data not provided				
	Severe adverse reactions (5)	5 incidence rate < 0.01%	1	1	1	1
		4 incidence rate 0.01% to <0.1%				
		3 incidence rate 0.1% to <1%				
		2 incidence rate 1% to <10%				
		1 incidence rate > 10%				
		0 ADR occurrence data not provided				
Special groups (multiple choices) (11)		2 available for children	0	0	0	0
		1 Available for the elderly (available 1, use with caution 0.5)	1	1	1	0.5
		1 Available to pregnant women (available in the first trimester 1, available in the second trimester 0.8, and available in the third trimester 0.5)	0	0	0	0
		1 Available for lactating women (available 1, use with caution 0.5)	0	0	0	0
		3 Abnormal liver function available (severe available 3, moderate available 2, mild available 1)	3	3	3	1
		3 Abnormal renal function is available (severe available 3, moderate available 2, mild available 1)	3	2	2	1
Adverse reactions caused by drug interactions (3)		3 No dosage adjustment required	1	1	1	1
		2 Need to adjust dosage				
		1 Prohibited to use at the same time				
Others (multiple choices) (3)		1 Adverse reactions are reversible	1	1	1	1
		1 No teratogenic, carcinogenic	0	0	0	0
		1 No specific medication warnings	1	1	1	1
Safety score			12	11	11	7.5

ADR = adverse reactions.

### 3.4. Economy score

The dosages referenced for the average daily treatment costs of the 4 CDK4/6 inhibitors in this assessment were derived from the drug instructions. Drug prices are sourced from the Guangzhou Drug Group Purchasing Platform, with a search date of July 25, 2023. Due to the unique health policy in China, abemaciclib and dalpiciclib are currently in the price confidentiality period, so their detailed prices are not released to the public for the time being. The drug information is detailed in Table 5.

**Table 5:** Drug price information.

Scheme number	Drug generic name	Product name	Specifications	Manufacturer	Unit price/bottle (box)	Dosage	Average daily treatment cost
1	Palbociclib	Ibrance	125 mg, 21* capsules/bottle	Pfizer Manufacturing Deutschland GmbH, Betriebsstatte Freiburg	CNY 4275. CNY 6	125 mg/time, once a day, stop for 1 wk after taking for 3 wk	CNY 152.7
2	Palbociclib	/	125 mg, 21* capsules/bottle	QILU PHARMACEUTICAL	CNY 3620	125 mg/time, once a day, stop for 1 wk after taking for 3 wk	CNY 129.29
3	Palbociclib	/	125 mg, 21* capsules/bottle	Jiangxi Shanxiang Pharmaceutical Co., Ltd	CNY 3780	125 mg/time, once a day, stop for 1 wk after taking for 3 wk	CNY 135
4	Palbociclib	AOPAIBO	125 mg, 21* capsules/bottle	Jiangsu Aosaikang Pharmaceutical Co., Ltd	CNY 3633	125 mg/time, once a day, stop for 1 wk after taking for 3 wk	CNY 129.75
5	Palbociclib	/	125 mg, 21* capsules/bottle	Beijing Tide Pharmaceutical Co., Ltd	CNY 3688	125 mg/time, once a day, stop for 1 wk after taking for 3 wk	CNY 131.71
6	Palbociclib	/	125 mg, 21* capsules/bottle	Hunan Kelun Pharmaceutical Co., Ltd	CNY 4275.6	125 mg/time, once a day, stop for 1 wk after taking for 3 wk	CNY 152.7
7	Palbociclib	/	125 mg, 21* capsules/bottle	Jiangsu Hansoh Pharmaceutical Group Co., Ltd	CNY 3799	125 mg/time, once a day, stop for 1 wk after taking for 3 wk	CNY 135.68
8	Ribociclib	Kispali	200 mg, 63* capsules/bottle	Novartis Singapore Pharmaceutical Manufacturing Pvt. Ltd.	CNY 13360	600 mg/time, once a day, stop for 1 wk after taking for 3 wk	CNY 477.14
9	Abemaciclib	Verzenio	150 mg, 14* piece/box	Lilly del Caribe Inc.	*	150 mg/time, twice a day, continuous use (do not stop)	CNY 170
10	Dalpiciclib	AIRUIKANG	150 mg, 21* piece/box	Jiangsu Hengrui Medicine Co., Ltd	*	150 mg/time, once a day, stop for 1 wk after taking for 3 wk	CNY 153.75

* Indicates that it is not displayed for Chinese policy reasons.

#### 3.4.1. Drugs with same generic name.

Seven drugs have the same generic name as palbociclib at 125mg. The average daily cost of treatment for the original palbociclib was CNY152.7, and the lowest average daily cost of treatment among palbociclib with the same generic name was CNY129.29. Based on the formula “assessed drug score = lowest average daily cost of treatment/average daily cost of treatment of the assessed drug × 3,” the score for palbociclib was 2.54 points. Ribociclib, abemaciclib, and dalpiciclib received a score of 3 because they are not yet available as drugs with the same generic name.

#### 3.4.2. Substitutable drugs for the main indications.

Among the 4 CDK4/6 inhibitors that were substitutable for the primary indication, palbociclib had the lowest average daily cost of treatment at CNY152.7, scoring 7 points. The remaining 3 CDK4/6 inhibitors ranked from lowest to highest in terms of the average daily cost of treatment were dalpiciclib, abemaciclib, and ribociclib. According to the formula “assessed drug score = lowest average daily cost of treatment/ average daily cost of treatment of the assessed drug × 7,” the scores of ribociclib, abemaciclib, and dalpiciclib were 2.24, 6.29, and 6.95, respectively.

In summary, the economy scores of palbociclib, ribociclib, abemaciclib, and dalpiciclib were 9.54 points, 5.24 points, 9.29 points, and 9.95 points, respectively, as shown in Table 6.

**Table 6:** Economy score results.

Economy (10)		Palbociclib	Ribociclib	Abemaciclib	Dalpiciclib
Drug with generic name (3)	3 Evaluation method: The drug with the lowest average daily treatment cost gets 3 points, Evaluation drug score = (minimum average daily treatment cost/average daily drug treatment cost) × 3.	2.54	3	3	3
Alternative drugs for main indications (7)	7 Evaluation method: the drug with the lowest average daily treatment cost gets 7 points, Evaluation drug score = (lowest average daily treatment cost/average daily drug treatment cost) × 7.	7	2.24	6.29	6.95
Economy score		9.54	5.24	9.29	9.95

### 3.5. Other attributes score

#### 3.5.1. National health policy.

Palbociclib, abemaciclib, and dalpiciclib are included in the National Health Insurance Catalog Category B reimbursement with no payment limitations, scoring 2 points. Ribociclib is not included in the National Health Insurance Catalog, scoring 1 point. None of the 4 CDK4/6 inhibitors are included in the National Essential Drug List, getting 1 point. Moreover, the 4 CDK4/6 inhibitors are not part of the national centralized procurement of drugs, so they are not scored.

#### 3.5.2. Manufacturers and global utilization.

Four CDK4/6 inhibitors were originator drugs, and all scored 1 point. According to the 2023 “Top 50 Global Pharmacological Companies” list published by *Pharmacological Executive* magazine in the US, Pfizer Manufacturing Deutschland GmbH, the manufacturer of palbociclib, is ranked 1st, scoring 1 point. Novartis Singapore pharmacological Manufacturing Pte. Ltd, the manufacturer of ribociclib, is ranked 4th, scoring 1 point. Lilly del Caribe Inc, the manufacturer of abemaciclib, is ranked 13th, scoring 0.8 points. Jiangsu Hengrui Pharmaceuticals Co. Ltd, the manufacturer of dalpiciclib, is ranked 43rd, scoring 0.2 points.

Palbociclib and abemaciclib are marketed in China, the US, Europe, and Japan, scoring 1 point. Ribociclib is marketed in China, the US, and Europe but not in Japan, scoring 0.5 points. Dalpiciclib is currently only available in China, so it is not scored.

To summarize, the results of the other attribute scores for palbociclib, ribociclib, abemaciclib, and dalpiciclib were 6, 4.5, 5.8, and 4.2, respectively, as shown in Table 7.

**Table 7:** Other attributes score results.

Other properties (10)		Palbociclib	Ribociclib	Abemaciclib	Dalpiciclib
National Health Insurance (3)	3 National Health Insurance Catalog Category A, and there are no payment restrictions	2		2	2
	2.5 National Health Insurance Catalog Category A, with payment restrictions				
	2 National Health Insurance Catalog Category B, no payment restrictions				
	1.5 National Health Insurance Catalog Category B, with payment restrictions				
	1 Not in the National Health Insurance Catalog		1		
National Essential Drugs (3)	3 National Essential Drugs, no* requirement	1	1	1	1
	2 National Essential Drug, with* requirement				
	1 Not in the National Essential Drug List				
National Centralized Procurement of Drugs (1)	1 National centralized procurement of selected drugs	0	0	0	0
Original research/reference/consistency evaluation (1)	1 Original drug/reference drug	1	1	1	1
	0.5 Generic Drugs Through Consistency Evaluation				
Production enterprise status (1)	1 The top 50 pharmaceutical companies in the world by sales/Top 100 companies in the pharmaceutical industry of the Ministry of Industry and Information Technology (1–10 of the world’s top 50 pharmaceutical companies by sales 1, 11–20 0.8, 21–30 0.6, 31–40 0.4, 41–50 0.2; Ministry of Industry and Information Technology Top 100 Enterprises in Pharmaceutical Industry 1–20 1, 21–40 0.8, 41–60 0.6, 61–80 0.4, 81–100 0.2)	1	1	0.8	0.2
Global usage (1)	1 Available in China, the United States, Europe, and Japan	1	0.5	1	0
	0.5 Available in China and other countries				
Scoring for other attributes		6	4.5	5.8	4.2

* Indicates that the drug should be used under the guidance of physicians with qualifications, and the durg monitoring and evaluation should be strengthened.

### 3.6. Comprehensive score results

Integrating the results of the 5 dimensions of pharmacological properties, efficacy, safety, economy, and other attributes of the 4 CDK4/6 inhibitors, the results of the combined scores of palbociclib, ribociclib, abemaciclib, and dalpiciclib were 78.04, 69.24, 78.09, and 72.15, respectively, as shown in Table 8. The top scorer was abemaciclib.

**Table 8:** Comprehensive score results.

Evaluation dimension	Palbociclib	Ribociclib	Abemaciclib	Dalpiciclib
Pharmacological properties	27.5	27.5	27	26.5
Efficacy	23	21	25	24
Safety	12	11	11	7.5
Economy	9.54	5.24	9.29	9.95
Other properties	6	4.5	5.8	4.2
Total score	78.04	69.24	78.09	72.15

## 4. Discussion

The composite scores showed that abemaciclib had the highest score of the 4 CDK4/6 inhibitors assessed, with palbociclib scoring only slightly lower than abemaciclib. The main reason is that both have sufficient clinical data and favorable safety and economic profiles. Scoring third was dalpiciclib, which had broadly similar scores to the top 2 regarding pharmacological properties, efficacy, and economy but was not superior in terms of safety. The safety score for dalpiciclib is low because there are no pharmacokinetic studies of dalpiciclib in patients with hepatic or renal impairment, and the use of dalpiciclib is not recommended in patients with moderate-to-severe hepatic or renal impairment. The lowest score was for ribociclib, due to its relatively high price, temporary classification as a Class II recommendation in China’s CSCO guidelines, and temporary exclusion from the National Health Insurance Catalog.

The issue of cost-effectiveness remains critical in oncology to ensure the sustainability of treatments.^[21]^ The cost-effectiveness of CDK4/6 inhibitors for the treatment of HR+/HER2− advanced breast cancer has been analyzed in some of the literature,^[22–27]^ but the results of the literature may be different from the results of the scores in this evaluation. The reason is that the results of cost-effectiveness analysis in other countries cannot be directly applied to the selection of CDK4/6 inhibitors by Chinese medical institutions. Furthermore, under the current national conditions and medical policies in China, in addition to cost-effectiveness, pharmacological properties, health insurance attributes, and the scope of centralized purchasing are all important criteria for medical institutions when selecting drugs.

This assessment provides a more objective data collection, integration, and comprehensive assessment based on the best available evidence for each attribute. However, some limitations remain: Some scoring rules and scores still need to be optimized. For example, in the scoring of efficacy indicators, since the 4 CDK4/6 inhibitors lacked head-to-head clinical trials and were scored only based on their respective phase III clinical trial data from the same control group, and since the *Rapid Guidelines* only stipulate that those with the best data on efficacy indicators will receive a total score, but do not stipulate specific scoring criteria and scores for the remaining medications, all of these may bias the scoring of this section. The results of this assessment are not static. As evidence from clinical studies of drugs continues to grow, guidelines are updated, and prices continue to change, assessment scores may change accordingly. Therefore, the drug assessment of CDK4/6 inhibitors still needs to be continuously updated and dynamically adjusted.

## 5. Conclusion

This assessment can provide a reference basis for Chinese medical institutions to select CDK4/6 inhibitors for treating HR+/HER2− advanced breast cancer and optimize the drug catalog. Pending updates on clinical studies, guideline recommendations, prices, and many other aspects of this assessment, the top 2 scoring abemaciclib and palbociclib may be prioritized for a recommendation based on the scoring results of this assessment. In practical application, medical institutions can combine the scoring results of this assessment and the actual situation of medical institutions to access or transfer out CDK4/6 inhibitor varieties rationally.

## Acknowledgments

We want to thank all the authors who participated in this study.

## Author contributions

**Conceptualization:** Hangye Gu, Zeyu Xie, Yong Chen.

**Data curation:** Hangye Gu, Yaqing Chen, Zeyu Xie, Yong Chen.

**Formal analysis:** Hangye Gu, Yaqing Chen, Zeyu Xie, Yong Chen.

**Funding acquisition:** Yong Chen.

**Investigation:** Hangye Gu, Yong Chen.

**Methodology:** Hangye Gu, Zeyu Xie, Yong Chen.

**Project administration:** Zeyu Xie, Yong Chen.

**Resources:** Yong Chen.

**Software:** Yaqing Chen, Yong Chen.

**Supervision:** Zeyu Xie, Yong Chen.

**Validation:** Yong Chen.

**Visualization:** Yong Chen.

**Writing – original draft:** Hangye Gu, Yong Chen.

**Writing – review & editing:** Hangye Gu, Yong Chen.
